# Acute Benefits of Acidified Milk Drinks with 10-g and 15-g Protein on Shifting and Updating Performances in Young Adults: A Randomized Controlled Trial

**DOI:** 10.3390/nu15020431

**Published:** 2023-01-13

**Authors:** Rui Nouchi, Laurie T. Butler, Daniel Lamport, Haruka Nouchi, Ryuta Kawashima

**Affiliations:** 1 Department of Cognitive Health Science, Institute of Development, Aging and Cancer (IDAC), Tohoku University, Sendai 980-8575, Japan; 2 Smart Aging Research Center, Tohoku University, Sendai 980-8575, Japan; 3 Faculty of Science and Engineering, Anglia Ruskin University, Cambridge CB1 1PT, UK; 4 School of Psychology & Clinical Language Sciences, University of Reading, Reading RG6 6AL, UK; 5 Department of Functional Brain Imaging, Institute of Development, Aging and Cancer, Tohoku University, Sendai 980-8575, Japan

**Keywords:** milk protein, acute benefit, shifting, updating, executive function, acidified milk

## Abstract

Background: Accumulating evidence has shown that protein-rich milk drinks can rapidly improve cognitive performance. However, the optimum doses of milk protein that are needed to improve cognitive function remain to be investigated. Objective: This study aimed to determine whether acidified milk drinks with 10-g and 15-g milk protein have acute benefits on key cognitive functions in healthy young adults. Design: In this double-blinded randomized control trial, 66 young adults were randomly assigned to one of three groups (0-g, 10-g, and 15-g milk protein groups). Key cognitive functions (processing speed, inhibition, shifting, updating, and working memory capacity) were assessed before and 15 and 60 min after the drink intake. Results: We found that the shifting performance improved at 15 min after intake of the acidified 10-g and 15-g milk protein drinks compared to intake of the 0-g milk protein drink, and this acute effect of the acidified 15-g milk protein drink lasted for 60 min. In addition, updating performance improved at 60 min after intake of the acidified 10-g and 15-g milk protein drinks compared to intake of the 0-g milk protein drink. Conclusion: Our findings suggest that the acidified 10-g and 15-g milk protein drinks have an acute benefit on shifting and updating performance in healthy young adults.

## 1. Introduction

Proteins are important for maintaining cognitive health [1]. For example, previous cohort studies have demonstrated that dietary protein intake is associated with better cognitive functions, such as memory, executive function, and processing speed, in healthy young and older adults [2,3]. Furthermore, randomized controlled trials (RCT) show that protein-rich drink or meal interventions can improve cognitive function in healthy young and older adults [4,5]. The results suggest that dietary proteins have an important role for cognitive health [6].

Proteins are widely available from foods such as meats, beans, and eggs. Milk protein is a particularly high-quality protein source [7]. Previous studies reported that increased intake of milk protein is associated with higher academic performance in young adults [8], a decreased risk of cognitive decline and dementia in the elderly [9], and improved cognitive function (e.g., attention and processing speed) in children [10], young [4,11,12], and older adults [5]. These findings suggest a beneficial effect of milk protein on cognition functioning.

Previous intervention studies reported that a single shot of milk protein intake improved cognitive functions [12,13]. The majority of cognitive benefits following milk consumption are observed 60 min post-prandially due to the rate of absorption. In general, milk is slowly absorbed due to gastric acid-induced coagulation [14]. It is plausible that cognitive performance could be improved if milk protein is absorbed more quickly. Acidification of milk using stabilizers and fermented cellulose is a possible mechanism to enable faster milk absorption [15]. Acidified milk can suppress aggregation and help maintain liquidity. The absorption rate of acidified milk has been shown to be faster than that of non-acidified milk (normal milk) [16,17]. Indeed, a recent study reported that an acidified milk protein drink enhanced mathematical calculation performance throughout the testing period (from 15 min to 120 min after ingestion) [15].

Although the previous study indicated the possibility that an acidified milk protein drink would improve cognitive performance [15], some issues remain unresolved. First, the previous study revealed that average cognitive performance for 2 h after the acidified milk protein drink was better compared to the placebo drink [15]. However, the previous study using the acidified milk protein drink did not compare cognitive performance at each test time point. Therefore, the time course of benefit of acidified milk intake on cognitive functions is unclear. Second, the previous study showed that acidified milk protein drink with 16-g milk protein improved cognitive performance in healthy young adults [15]. It still remains unclear whether lower doses of protein in an acidified milk drink would affect an improvement of cognitive performance. The recent study showed that the acidified milk drink with 10-g protein rapidly increased essential amino acid such as leucine at 30 min after intake [17,18]. Previous studies have shown that these amino acids play an important role in maintaining and improving cognitive function [6,19,20,21]. Therefore, it is possible that a lower-dose milk protein (10-g), delivered as part of an acidified milk drink, would also have a positive effect on cognitive performance. Third, previous studies found that non-acidified milk protein drinks significantly improved a wide range of cognitive functions, such as calculation, attention, processing speed, and working memory [11,12,13,22]. However, no study has investigated the beneficial effects of acidified milk protein drinks on a wide range of cognitive performance tasks. Fourth, previous studies have shown that fasting glucose affected acute cognitive improvement [22,23,24]. One previous study using the non-acidified milk protein drink revealed an improvement in working memory performance most clearly in those with higher fasting glucose [22]. However, the only previously published study using an acidified milk protein drink did not measure fasting glucose level [15]. It is not clear whether individual differences in glucoregulation would affect an acute cognitive benefit after the acidified-milk protein drink.

In this study, we used acidified 10-g and 15-g milk protein drinks. There were several reasons for these doses. Firstly, previous studies indicate that intake of an acidified 10-g milk protein drink can rapidly increase essential amino acid levels [17,18]. Furthermore, amino acid levels have been positively associated with cognitive performance [6,19,20,21]. Therefore, we predicted that the acidified 10-g milk protein drink would have positive effects on cognitive function. Secondly, a previous study with an acidified milk protein drink (16-g milk protein and 28-g carbohydrate) and a placebo drink (0-g milk protein and 42-g carbohydrate) had large differences in carbohydrate content between different drinks. Carbohydrate content can affect cognitive performance [25]. Therefore, to address this limitation in this study, we used the same amount of glucose among the three drinks. Finally, Japanese Dietary Reference Intakes by the Ministry of Health, Labor, and Welfare recommend an average 15-g protein per meal a day (https://www.mhlw.go.jp/file/06-Seisakujouhou-10900000-Kenkoukyoku/Overview.pdf, accessed on 4 January 2023). Therefore, we chose the acidified 0-g, 10-g, and 15-g milk protein drinks in this study.

To address the abovementioned unresolved issues, we conducted an RCT using two different doses of an acidified milk protein drink (10-g and 15-g) and compared this to a 0-g protein control drink. We measured key cognitive domains, such as processing speed, executive function (inhibition, shifting, and updating), and working memory capacity, at 15 and 60 min after consumption. In addition, we assessed the fasting glucose and glucose at each time point. 

The purpose of this study was to investigate the time course of the acute benefits of acidified 10-g and 15-g milk protein drinks on key cognitive functions. In this study, we recruited young healthy adults. Previous research using an acidified milk protein drink has shown the acute improvement of simple calculation performance in healthy young adults [15]. Therefore, we expected that acidified 10-g and 15-g milk protein drinks can improve performance on other cognitive domains in healthy young adults. We hypothesized that intake of drinks containing 10-g and 15-g acidified milk protein would improve cognitive functioning compared to the intake of drinks without milk protein. In addition, we expected that the acute benefit of the acidified milk protein drinks would be observed at 15 min, which coincides with the anticipated increased in amino acid levels within 30 min after intake of an acidified milk protein drink [16,17]. 

## 2. Materials and Methods

### 2.1. Design and Setting of This RCT

This RCT was conducted in Sendai city, Japan, and the Ethics Committee of Tohoku University Hospital approved the study protocol (protocol code 2018-2-276). This study was conducted in accordance with the principles outlined in the Declaration of Helsinki. The RCT was registered in the University Hospital Medical Information Network Clinical Trial Registry (UMIN000035796).

To determine the acute effect of acidified milk protein drinks on cognitive function in healthy young adults, we conducted a parallel groups double-blinded RCT. Both participants and researchers were blinded to condition. The primary outcome measure was a Plus-Minus task. Other cognitive functions and mood states (described below) were secondary outcome measures. The Consolidated Standards of Reporting Trials statement (http://www.consort-statement.org/home/, accessed on 4 January 2023, see Supplementary Materials Table S1) was used to report the study findings.

### 2.2. Participants

We recruited undergraduate and graduate students using online advertisements at the university. Inclusion and exclusion criteria were written on flyers. Based on an earlier intervention study [15], we used the following inclusion and exclusion criteria: (1) native Japanese speakers with normal vision; (2) not having food allergy; (3) 20–30 years old; (4) not having a regular habit of consuming alcohol; (5) not using medications known to interfere with cognitive functions (including benzodiazepines, antidepressants, or other central nervous agents); (6) no history of any disease known to affect the central nervous system, such as heart disease, multiple sclerosis, Parkinson’s disease, stroke, diabetes, or mental diseases; (7) not using implantable medical devices, such as a pacemaker; and (8) not receiving artificial dialysis. In addition, participants who were participating in other cognition-related intervention studies or those who were pregnant were excluded.

**Figure 1:**
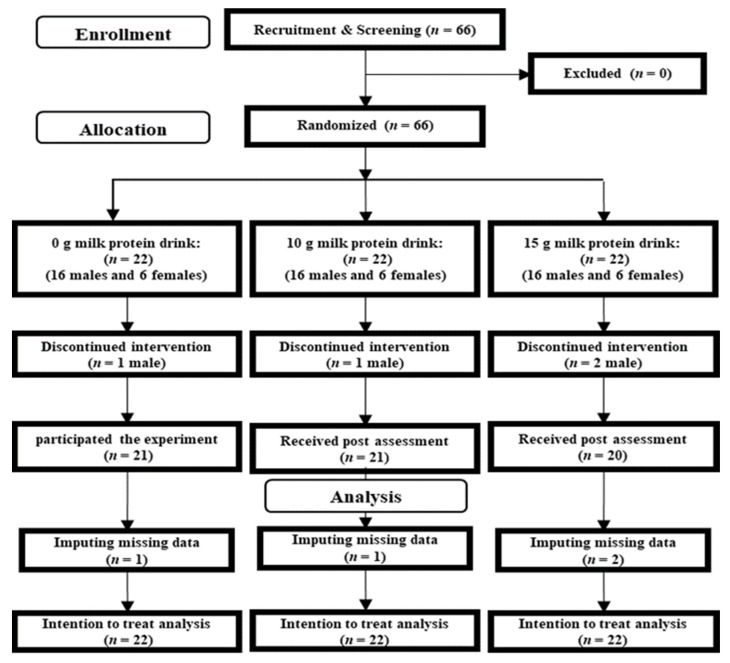
CONSORT diagram.

A total of 66 participants contacted the research group via e-mail (Figure 1). After an explanation of the experimental purpose and procedure, we obtained informed consent from each participant. A researcher then checked whether the participants were eligible to participate in this study, and no patients were excluded at this time. The 66 participants were randomly assigned to the 15-g, 10-g, and 0-g milk protein groups. Four participants dropped out between screening and the start of the experiment. Table 1 presents baseline characteristics of all participants (*n* = 62; 44 men and 18 women).

### 2.3. Sample Size

We calculated the sample size using a simulation with the SIMR package (version 1.0.4) in R (R Core Team, Vienna, Austria) [26]. The sample size was calculated based on the primary outcome of the plus-minus task. A previous study using the simple adding task, which required participants to add single digits, reported a large effect (*dz* = 2.925) of 16-g acidified milk protein intake relative to placebo (0-g milk protein) at 15 min after milk protein intake [15]. Based on this and other evidence, we expected a medium to large effect at 15 min and 60 min after 15-g acidified milk protein intake. To calculate the sample size, we used a generalized linear mixed model (GLMM) model for the plus-minus task with *α* = 0.05 and 0.80 power. We used drinks (15-g, 10-g, and 0-g (placebo) milk proteins), time (before and 15 min and 60 min after the drink intake), age, and sex as fixed effects. Participants were classified as a random effect. Based on 1000 data simulations, the sample size was set to 66 (22 participants per group). 

### 2.4. Randomization

To assign the 66 interested participants randomly to one of the three drink groups, we used an online program for randomization (http://www.graphpad.com/quickcalcs/index.cfm, accessed on 4 January 2023). R.N. conducted the randomization method. We stratified participants according to sex to reduce the impact of sex effects. We used blocked randomization (block size, 6) with an allocation ratio of 1:1:1. 

### 2.5. General Procedure

The RCT design is shown in Figure 1. Before the experimental day, participants provided informed consent. At screening, inclusion/exclusion criteria were checked. Verbal and non-verbal general intelligence was measured using the Raven metrics test [27] and the Japanese reading ability test [28]. Participants were then randomly assigned to 15-g, 10-g, and 0-g milk protein drink groups.

**Figure 2:**
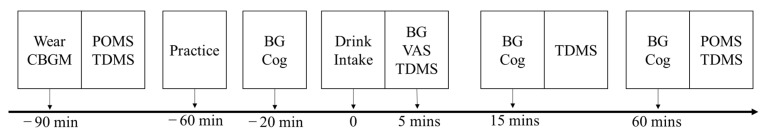
Experimental procedure. Note: CBGM = continuous blood glucose monitoring; POMS = profile of mood state; TDMS = two-dimensional mood scale; BG = blood glucose; Cog = cognitive function assessments; VAS = visual analog scale for drinking.

On the day before the experimental day, all participants were instructed to not consume alcohol or engage in exercise. In addition, we asked participants to refrain from consuming any food or drink (except water) after 22:00, and sleep before 24:00. On the experimental day (Figure 2), participants came to the experimental room before 9:00. A continuous glucose monitoring sensor was applied to the back of the upper arm (see 2.10 Measurement of Blood Glucose Levels section). Then, participants performed the battery of cognitive functional tests (see 2.7 Cognitive Functional Assessments section) and rated their current mood states (see 2.8 Mood State Measure section). Participants underwent cognitive assessments before intake of the acidified milk protein drinks. Participants rated their current mood and answered subjective sweetness, sourness, bitterness, and a general preference about the drink using visual analog scales. Subsequent cognitive and mood assessments were performed at 15 min and 60 min post-consumption of the drink intake. 

### 2.6. Milk Protein and Placebo Drinks

We prepared 15-g milk protein, 10-g milk protein, and 0-g milk protein drinks (400 mL). The drinks could not be identified from their appearance. Table 2 lists the composition of each drink. Participants consumed one of these drinks. The 15-g and 10-g milk protein drinks were acidified [15]. The drinks were manufactured by the R&D Division of Meiji Co., Ltd. (Tokyo, Japan). Milk protein concentrate was used to make the 15-g and 10-g milk protein drink, which matched previous studies [15]. The company provided drinks with three different 3-digit labels (e.g., 245) in order to maintain double blinding. The researchers did not know the composition of each drink until the statistical analysis was completed. 

### 2.7. Cognitive Functional Assessments

We assessed the performance of executive functions (shifting, inhibition, and updating), processing speed, and working memory capacity before and 15 and 60 min after drinking. All cognitive assessments were performed based on computer versions built by PsychoPY (version 3.1.2, Open Science Tools, Nottingham, UK) [29]. It took approximately 10 min to conduct all cognitive assessments. For shifting performance, we used the plus-minus task [30,31]. The plus-minus task was developed to measure the speed and accuracy of shifting from one rule to another for individuals. This task had three blocks (add, subtract, and mix) with 20 two-digit numbers. In the first block (add block), participants were asked to add three to each presented number (e.g., 42 + 3). In the second block (subtract block), participants were asked to subtract 3 from each presented number (e.g., 72 − 3). In the final block (mix block), participants were asked to shift between adding 3 to and subtracting 3 from each number. The shifting cost (reaction time) was computed by subtracting the average reaction time in the added and subtracted blocks from the reaction time in the mixed block.

For inhibition performance, we used the stroop test (ST). The following procedure was based on the paper–pencil version ST [32]. In the ST, the target was presented to the left of most of the six words. The target was a color word that was printed in another color (e.g., ‘red’ is printed in blue letters), and the other five words had different colors. Participants were asked to select as many correct words (the color words that were the same as the target) as possible within 60 s. The primary measure for this task was the number of correct items. 

To measure updating performance, we used an adapted version of the verbal running working memory updating task [33,34]. We used a list of four, six, eight, and ten digits. There were two trials for each list (a total of eight lists). The lists were presented in randomized order. The participants were asked to serially answer the last four items of each list. Before starting the task, participants were instructed that the four-, six-, eight-, and ten-digit lists were presented in random order. However, they were not informed of the list length beforehand. The outcome measure of this test was accuracy.

For processing speed, we used the symbol digit coding task [35,36]. In this task, participants were required to translate nine different symbols into nine digits (1–9) as quickly as possible in 60 s. A coding key of nine symbol–digit pairs was presented at the top of the screen. The primary measure of this test was the number of correct answers.

To measure working memory capacity, we used the visual digit span backward, which is a modified auditory digit span backward [37,38]. Participants viewed digit sequences and had to recall them in the reverse order. Participants typed their answers using a keyboard. We used 4 four-digit to 10-digit sequences. Participants viewed a sequence of visual digits (starting a list of three digits), where each digit was presented for 1 s. After the recall instruction cue, participants were asked to recall the digit sequence and type the answer into the presented text box. The participants took two sets in each digit sequence. If one of the responses at the same digit sequence was correct (in digits and presentation order), the participant moved up to the next level (e.g., level 3). If both responses in the same digit sequence were incorrect, the same level was presented again. If a consecutive error occurred, the participant moved back down to a lower level and started all over again. The outcome of this test was the maximum number of digits.

### 2.8. Mood State Measure

We used a short version of the Profile of Mood State Second Edition [39,40] and Two-Dimensional Mood Scale (TDMS) [41] to assess the change in mood state, as described in our earlier report [42]. The Profile of Mood State Second Edition has 7 subscales (tension–anxiety, depression–rejection, anger–hostility, vigor–activity, fatigue–inertia, confusion–bewilderment, and friendliness) with 5-point scales (from 0 (not at all) to 5 (extremely)). It has a total of 35 items and can measure the current mood states. The score range of each subscale range was from 0 to 25.

In addition, we measured acute mood changes using the TDMS, which was developed to measure momentary mood states [41]. The TDMS has eight items (Energetic, Lively, Lethargic, Listless, Relaxed, Calm, Irritated, and Nervous) with a 6-point Likert scale (from 0 (not at all) to 5 (extremely)). We used the score in each item as outcome measure [41]. Participants rated the TDMS before the pre-cognitive assessment and immediately, 15 min, and 60 min after intake of the drink.

### 2.9. Visual Analog Scales for Drinks

Previous studies have reported that taste affected cognitive processing [43,44]. Therefore, we asked participants to rate subjective sweetness, sourness, bitterness, and a general preference for the intake drink. Participants checked a mark at a point on a 10 cm visual analog scale immediately after intake of the drink. The anchor points for visual analog scale ranged from do not feel at all (scored 0) to strongly feel (scored 10).

### 2.10. Measurement of Blood Glucose Levels

Blood glucose levels were measured using a free-style Libre continuous glucose monitoring system (FGM, Abbott Diabetes Care, Inc., Alameda, CA, USA) [45]. This glucose monitoring system works via a sensor inserted under the skin of the upper arm through an applicator. We measured the blood glucose levels (1) before the drink intake, (2) immediately after the drink intake, (3) 15 min the drink intake, and (4) 60 min after the drink intake. Changes in blood glucose levels are shown in Table 3.

### 2.11. Analysis

All analyses were conducted using R software (version 3.53, R Core Team, Vienna, Austria). All participants were included based on the intention to treat (ITT) principle. We imputed missing values (m = 20) using all variables of cognitive function performance, age, and sex. To impute missing dates, we used the “mice” function in the mice package [46]. We used predictive mean matching because the predictive mean matching method in multiple imputations can work even if the sample size is small [47]. In addition, we used a permutation analysis of variance with the “aovp” function in the lmPerm package (http://cran.r-project.org/web/packages/lmPerm/index.html, accessed on 4 January 2023) to determine whether baseline measurements (demographic factors, the blood glucose levels, cognition, and mood) and subjective feeling for the drink differed between groups. 

Moreover, to investigate change of the blood glucose levels among groups, we conducted GLMM with “lmer” in the lme4 package (https://cran.r-project.org/web/packages/lme4/index.html, accessed on 12 January 2023) and “with” in the mice package (https://cran.r-project.org/web/packages/mice/index.html, accessed on 12 January 2023) and pooled all the results with “pool” in the mice package. In the third GLMM model, we used drinks (15-g, 10-g, and 0-g milk proteins), time (before the drink intake, immediately after the drink intake, 15 min after the drink intake, and 60 min after the drink intake), age, and sex as fixed effects. Participants were used as a random effect. In addition, to investigate an association between cognitive performance and glucose levels, we conducted additional Spearman’s rank correlation analysis among glucose level and cognitive performance.

For the main analysis, we analyzed cognitive functions using a GLMM. In the GLMM, we used drinks (15-g, 10-g, and 0-g milk proteins), time (before the drink intake, 15 min after the drink intake, and 60 min after the drink intake), age, and sex as fixed effects. Participants were used as a random effect. In these GLMM analyses, we were interested in the interaction between the factors of drinks and time. Significance was inferred for multiple comparison methods *(p* < 0.05). We used false discovery rate correction methods [48] to adjust all pooled *p*-values using “*p*.adjust” in R (version 3.53, R Core Team, Vienna, Austria). If we found an interaction effect in GLMMs, we investigated a simple main effect using the Tukey correction method with “lsmeans” in the lsmean package (https://cran.r-project.org/web/packages/lsmeans/index.html, accessed on 12 January 2023).

We also conducted an additional analysis for mood state measurements. We used a similar GLMM model to cognitive functions’ analysis. We used the false discovery rate correction to adjust *p* values.

## 3. Results

First, we investigated the baseline differences and compared subjective ratings for the drinks among the groups using analysis of variance (permutation ANOVA). There were no significant effects of age (*F* (1, 64) = 0.00, *p* = 0.92), height (*F* (1, 64) = 0.47, *p* = 0.82), weight (*F* (1, 64) = 0.11, *p* = 0.76), Raven (*F* (1, 64) = 1.26, *p* = 0.21), or Japanese Reading Test (JART) (*F* (1, 64) = 1.39, *p* = 0.57) among drink groups at baseline (Table 1). In addition, for blood glucose we did not find any significant main effect of drink group (*F* (1, 64) = 0.49, adjusted *p* = 0.486) nor an interaction effect between drink group and time (*F* (3, 192) = 0.222, adjusted *p* = 0.880) (Table 3). There were no significant correlations between fasting glucose levels and cognitive performance (Table 4). We did not find any significant differences in subjective rating for the drinks between the drink groups (Sweetness: *F* (1, 64) = 0.00, *p* = 0.963, Sourness: *F* (1, 64) = 0.06, *p* = 0.808, Bitterness: *F* (1, 64) = 0.05, *p* = 0.830, General preference: *F* (1, 64) = 1.29, *p* = 0.261: Table 5)).

We then performed GLMM analysis with 3 (drinks: 15-g, 10-g, and 0-g milk protein) by 3 (time: before the drink intake, 15 min after the drink intake, and 60 min after the drink intake) for each cognitive assessment (Table 6). We found a significant interaction effect between drink and time factors on shifting performance (*F* (2, 128) = 7.054, adjusted *p* = 0.016). Post-hoc analysis revealed better shifting performance in the 15-g and 10-g milk protein groups compared to the 0-g milk protein group at 15 min after drink intake (Table 5). In addition, the 15-g milk protein group performed better than the 0-g milk protein group at 60 min after the drink intake. We also found a significant interaction effect between drink and time factors on update performance *(F* (2, 128) = 5.02, adjusted *p* = 0.020). Post-hoc analysis revealed better updating performance in the 15-g and 10-g milk protein groups than in the 0-g milk protein group at 60 min after the drink intake (Table 5). However, we did not find other significant interaction effects on processing speed (*F* (2, 128) = 0.413, adjusted *p* = 0.828), working memory capacity (*F* (2, 128) = 0.730, adjusted *p* = 0.806), and inhibition performance (*F* (2, 128) = 0.135, adjusted *p* = 0.874). 

For the additional analysis of mood state, we also did not find any significant interactions on POMS (tension–anxiety: *F* (1, 64) = 0.16, adjusted *p* = 0.885, depression–rejection: *F* (1, 64) = 0.38, adjusted *p* = 0.885, anger–hostility: *F* (1, 64) = 0.12, adjusted *p* = 0.885, vigor–activity: *F* (1, 64) = 2.72, adjusted *p* = 0.541, fatigue–inertia: *F* (1, 64) = 0.00, adjusted *p* = 0.998, confusion–bewilderment: *F* (1, 64) < 0.01, adjusted *p* = 0.998, and friendliness: *F* (1, 64) = 1.84, adjusted *p* = 0.541) (Table 7). In addition, we did not find any significant interactions on TDMS (Energetic: *F* (3, 192) = 1.78, adjusted *p* = 0.541, Lively: *F* (3, 192) = 2.10, adjusted *p* = 0.541, Lethargic: *F* (3, 192) = 0.799, adjusted *p* = 0.885, Listless: *F* (3, 192) = 0.53, adjusted *p* = 0.885, Relaxed: *F* (3, 192) = 0.54, adjusted *p* = 0.885, Calm: *F* (3, 192) = 0.58, adjusted *p* = 0.885, Irritated: *F* (3, 192) = 0.38, adjusted *p* = 0.885, and Nervous: *F* (3, 192) = 1.85, adjusted *p* = 0.541) (Table 8).

## 4. Discussion

This study investigated whether 10-g and 15-g milk protein drinks, respectively, have acute effects on a wide range of cognitive functions in healthy young adults. There were two main findings. First, shifting performance measured by the plus-minus task improved at 15 min after the intake of both the 10-g and 15-g milk protein drinks compared to the 0-g milk protein drink; in addition, we found that the acute effect of the 15-g milk protein drink lasted for 60 min, but the 10-g milk drink did not. Second, updating performance improved at 60 min after intake of the 10-g and 15-g milk protein drinks compared to intake of the 0-g milk protein drink. Our findings suggest that milk protein intake rapidly improves cognition performance in young adults.

We found acute beneficial effects of both 10-g and 15-g milk protein on shifting performance at 15 min after intake. These findings are consistent with those of previous studies with a larger sample cohort and long-term intervention studies. For example, cohort studies demonstrated that dietary protein intake was associated with better shifting performance in older adults [2,49], and a longitudinal intervention study found that 8.8 g milk protein intake improved shifting performance in young children after 4.5 months of intervention [10]. Notably, our study is the first to demonstrate acute beneficial effects of milk protein on shifting performance in healthy young adults. However, one previous study showed that 8-g or 13-g milk protein drinks did not improve shifting performance at 60 min after the drink intake in young children [50]. Notably, however, the previous study used a normal milk protein drink [50], while our study used an acidified milk protein drink. Therefore, our study was able to determine the acute beneficial effects of 10-g and 15-g milk protein.

In addition, we found that updating performance was improved at 60 min after intake of the 10-g and 15-g milk protein drinks. This result is consistent with previous evidence. For example, a previous study demonstrated improved updating performance in young adults with higher fasting glucose levels after intake of the non-acidified milk protein drink [22]. However, we did not find any significant relationships between fasting glucose levels and updating performance (Table 4). Therefore, our study expanded the previous finding to demonstrate the acute beneficial effect of milk protein intake on the updating performance in healthy young adults, regardless of the fasting glucose level. It is important to note that the previous and current studies used different updating tasks. The previous study used a running memory continuous performance task, which is a speed measure of updating performance (e.g., reaction time). In the running memory continuous performance task, the stimulus was sequentially displayed on a computer screen; participants were required to judge whether the displayed stimulus matched the preceding stimulus, and they updated every stimulus and maintained one stimulus during the task. On the other hand, we used the verbal running working memory updating task, which required participants to update every stimulus and maintain four stimuli during the task. The verbal running working memory task focused on accuracy (total number of correct answers). Therefore, the verbal running working memory task would be more difficult than the running memory continuous performance task. It is possible that the different task difficulty levels might have led to different results. Specifically, an easier updating task might have revealed acute benefits of the milk protein drink at an earlier time, such as 15 min after the drink intake. 

Moreover, we did not find any significant improvements in inhibition, processing speed, or working memory capacity after milk protein intake. These results are consistent with those of previous studies. For example, previous studies showed that milk protein intake did not have any significant acute benefits on the inhibition measured by the Stroop task [15] and Go/no-Go [22], processing speed measured by the Trail Making Test A [51], and working memory capacity measured by the digit span backward task [50]. These findings suggest that milk protein intake does not have beneficial effects on inhibition, processing speed, or working memory capacity performance. 

The current study did not investigate the mechanisms underlying the acute beneficial effects of acidified milk protein intake on cognitive function. Milk protein contains a high proportion of amino acids [52], and the acidified milk drink also contains amino acids [17]. It is possible that changes in amino acid levels would have a critical role in the acute beneficial effects of acidified milk proteins. Indeed, accumulating studies have demonstrated that amino acids play an important role in maintaining and improving cognitive function [6,19,20,21]. For example, a 12-week essential amino acid intake intervention improved performances on the Trail-Making Test B, which is one of the executive functional measurements, in healthy middle-aged and older adults [20]; medium-chain triglycerides with leucine and vitamin D supplementation improved general cognitive performance, as measured by Mini Mental State Examination (MMSE), in frail older adults [21]; the acidified milk protein drink with 10-g protein rapidly increased the plasma levels of amino acids, especially leucine [17]. Moreover, it should be noted that various amino acids are known to be precursors of key brain neurotransmitters [53]. For example, tryptophan is the precursor of serotonin; tyrosine and phenylalanine are precursors of dopamine, noradrenaline, and adrenaline [54]; branched-chain amino acids, such as leucine, isoleucine, and valine, can also be converted into glutamate [55]. Leucine is an important donor for glutamate synthesis in the brain [56], and serotonin, dopamine, and glutamate levels are positively associated with cognitive performance [57,58]. Based on previous evidence, we hypothesized that (1) the acidified milk protein drinks would be quickly absorbed [18] and lead to an increase in amino acid levels in the body [14,17,59], (2) amino acid levels in the brain are increased after milk protein intake because several amino acids can pass through the brain–blood barrier [60], and (3) amino acids can be converted to neurotransmitters. Increased levels of neurotransmitters (serotonin, dopamine, or glutamate) could improve shifting and updating performances in executive functions.

This study has some limitations. First, we did not assess cognitive performance at further intervals beyond 60 min after milk protein intake. Thus, it is still unclear how long the benefits of the milk protein drink will last. A previous study showed the acute benefit of milk protein 120 min and 240 min after consumption of a high-protein meal [11]. In the future, it would be important to investigate whether a milk protein drink would improve cognitive performance within a few hours. Second, we did not evaluate any biological measurements (e.g., blood examinations, brain imaging, or physiological measurements). Further studies are needed to assess biological factors to reveal the mechanisms underlying the acute benefits of milk protein drinks on cognitive performance.

## 5. Conclusions

In summary, we investigated the acute benefits of acidified milk protein drinks on cognitive performance in healthy young adults. We found improvements in executive function (specifically shifting and updating performance) at 15 and 60 min after intake of acidified 10-g and 15-g milk protein drinks which were not observed following a 0-g milk protein drink. From this study, it is difficult to conclude how much milk protein is necessary to improve cognitive functions. The findings suggest that the acidified 10-g and 15-g milk protein intake can have acute benefits on executive functions in young adults. This finding could have relevance to clinical situations, given that executive functions decline in aging and clinical populations [61,62]. Future research could investigate whether acidified milk protein can improve executive functions in clinical populations.

## Figures and Tables

**Table 1:** Characteristics of participants.

		0-g Milk Group	10-g Milk Group	15-g Milk Group	*p* Value
Age	M	22.38	22.56	21.78	0.92
	SD	(1.75)	(1.76)	(1.66)	
Height	M	169.51	167.52	169.47	0.82
(cm)	SD	(6.69)	(9.61)	(8.06)	
Weight	M	61.21	60.52	59.26	0.76
(kg)	SD	(9.08)	(10.06)	(10.57)	
Japanese Reading Test	M	20.76	21.78	21.11	0.21
(score)	SD	(2.68)	(2.69)	(2.48)	
Raven’s progressive matrix test	M	29.76	28.67	29.63	0.57
(score)	SD	(2.41)	(2.22)	(3.85)	

Note: 0-g milk group = 0-g milk protein drink group, 10-g milk group = 10-g milk protein drink group, 15-g milk group = 15-g milk protein drink group, M = mean, SD = standard deviation.

**Table 2:** Composition of each drink.

Drink	0-g Milk Protein Drink Group	10-g Milk Protein Drink Group	15-g Milk Protein Drink Group
Basic ingredients (%)			
Milk protein	0	3.1	4.6
Glucose	4.6	4.6	4.6
Gelling agents	1.6	1.6	1.6
Sweetener	0.01	0.01	0.01
Flavoring agent	0.05	0.05	0.05
Acidifier	0.3	0.6	0.6
Water	93.5	90.1	88.6
Total	100	100	100
Nutrient			
Protein (g)	0.1	10.2	15.1
Fat (g)	0	0.2	0.3
Carbohydrate (g)	22.5	23.3	23.7
Dietary fiber (g)	1.3	1.3	1.3
Ash content (g)	0.5	1.3	1.7
Sodium (mg)	120	120	126
Energy (kcal)	92.7	138.3	160.3

**Table 3:** Means of blood glucose levels (mg/dL) before and after drink intake.

	Before the Drink Intake	Immediately after the Drink Intake	At 15 min after the Drink Intake	At 60 min after the Drink Intake
0-g milk group	82.21 (7.51)	80.74 (8.66)	83.42 (9.84)	89.40 (9.49)
10-g milk group	78.22 (10.60)	76.56 (8.65)	79.83 (9.87)	87.00 (14.15)
15-g milk group	81.95 (11.33)	79.58 (10.09)	81.26 (12.67)	86.74 (14.38)

Note: 0-g milk group = 0-g milk protein drink group, 10-g milk group = 10-g milk protein drink group, 15-g milk group = 15-g milk protein drink group. Standard deviation is shown in brackets.

**Table 4:** Spearman’s rank correlations among the fasting glucose levels and cognitive performance before and after drink intake.

	Before the Drink Intake	At 15 min after the Drink Intake	At 60 min after the Drink Intake
Digit symbol coding			
0-g milk group	0.01	−0.25	−0.19
10-g milk group	0.05	−0.18	−0.13
15-g milk group	0.28	0.08	−0.13
Stroop task			
0-g milk group	0.06	0.23	0.24
10-g milk group	−0.07	−0.24	−0.33
15-g milk group	0.08	0.06	0.30
Plus-minus task			
0-g milk group	0.00	0.02	0.05
10-g milk group	−0.21	0.37	0.06
15-g milk group	−0.20	0.30	−0.07
Verbal running working memory			
0-g milk group	0.15	0.13	0.25
10-g milk group	−0.16	−0.17	−0.34
15-g milk group	−0.19	−0.07	−0.10
Digit span backward			
0-g milk group	0.37	−0.07	0.37
10-g milk group	0.19	−0.13	0.02
15-g milk group	0.11	−0.09	−0.18

Note: 0-g milk group = 0-g milk protein drink group, 10-g milk group = 10-g milk protein drink group, 15-g milk group = 15-g milk protein drink group.

**Table 5:** Means of subjective rating of sweetness, sourness, bitterness, and general preference for each drink.

	0-g Milk Group	10-g Milk Group	15-g Milk Group
Sweetness	5.98 (2.32)	5.99 (2.26)	6.08 (2.34)
Sourness	5.42 (2.15)	5.18 (3.06)	5.48 (2.31)
Bitterness	0.71 (0.96)	0.77 (1.03)	0.88 (1.67)
General Preference	6.27 (2.02)	5.29 (2.42)	6.36 (2.55)

Note: 0-g milk group = 0-g milk protein drink group, 10-g milk group = 10-g milk protein drink group, 15-g milk group = 15-g milk protein drink group. Standard deviation is shown in brackets.

**Table 6:** Cognitive function before and 15 and 60 min after drink intake.

	Before the Drink Intake		At 15 min after the Drink Intake		At 60 min after the Drink Intake	
	M	SD	M	SD	M	SD
Digit symbol coding (total correct answers)						
0-g milk group	34.91	(4.99)	35.18	(4.56)	36.27	(4.91)
10-g milk group	33.91	(5.29)	33.91	(4.68)	36.05	(4.92)
15-g milk group	33.09	(5.16)	35.14	(4.22)	35.45	(4.86)
Stroop task (total correct answers)						
0-g milk group	62.57	(4.18)	63.14	(3.34)	64.95	(3.26)
10-g milk group	62.61	(4.22)	63.39	(4.29)	63.00	(5.19)
15-g milk group	60.79	(2.48)	61.26	(3.85)	61.21	(2.65)
Plus-minus task (msecs)						
0-g milk group	291	(283)	299	(220)	265	(140)
10-g milk group	293	(499)	49 *	(151)	67	(139)
15-g milk group	305	(373)	46 *	(159)	47 *	(143)
Verbal running working memory task (Accuracy (%))						
0-g milk group	0.73	(0.16)	0.73	(0.15)	0.76	(0.13)
10-g milk group	0.73	(0.19)	0.86	(0.12)	0.90 *	(0.13)
15-g milk group	0.75	(0.17)	0.84	(0.17)	0.91 *	(0.12)
Digit span backward (number of digits)						
0-g milk group	6.55	(1.82)	6.45	(1.84)	7.45	(1.60)
10-g milk group	6.00	(1.20)	6.64	(2.26)	7.59	(1.56)
15-g milk group	6.23	(1.51)	6.73	(2.27)	7.00	(2.07)

Note: 0-g milk group = 0-g milk protein drink group, 10-g milk group = 10-g milk protein drink group, 15-g milk group = 15-g milk protein drink group, M = mean, SD = standard deviation. * = significant differences compared to 0-g milk protein drink group.

**Table 7:** Profile of mood state scores before and after the experiment.

	Before Experiment		After Experiment	
	M	SD	M	SD
Anger–Hostility				
0-g milk protein drink-group	2.76	(3.62)	1.67	(2.13)
10-g milk protein drink-group	2.17	(3.45)	1.44	(2.33)
15-g milk protein drink-group	2.06	(2.86)	1.94	(3.86)
Confusion–Bewilderment				
0-g milk protein drink-group	4.33	(2.97)	3.67	(2.42)
10-g milk protein drink-group	4.56	(3.71)	3.94	(3.11)
15-g milk protein drink-group	4.00	(3.48)	3.17	(2.92)
Depression–Dejection				
0-g milk protein drink-group	3.95	(3.85)	3.19	(3.70)
10-g milk protein drink-group	2.56	(2.18)	1.72	(2.42)
15-g milk protein drink-group	2.22	(2.82)	2.06	(3.21)
Fatigue–Inertia				
0-g milk protein drink-group	5.38	(3.61)	4.95	(3.35)
10-g milk protein drink-group	5.83	(4.79)	4.94	(3.40)
15-g milk protein drink-group	4.39	(4.09)	4.89	(4.34)
Tension–Anxiety				
0-g milk protein drink-group	5.62	(2.77)	4.00	(2.65)
10-g milk protein drink-group	5.22	(3.64)	3.22	(2.67)
15-g milk protein drink-group	5.17	(4.20)	3.00	(3.27)
Vigor–Activity				
0-g milk protein drink-group	5.95	(3.06)	5.14	(4.05)
10-g milk protein drink-group	6.44	(3.99)	4.56	(3.55)
15-g milk protein drink-group	6.33	(3.58)	5.78	(3.56)
Friendliness				
0-g milk protein drink-group	10.95	(4.14)	11.33	(5.00)
10-g milk protein drink-group	9.94	(4.65)	9.80	(4.60)
15-g milk protein drink-group	11.33	(3.63)	10.33	(4.12)

Note: M = mean, SD = standard deviation.

**Table 8:** Two-dimensional mood scale scores before and 15 and 60 min after drink intake.

	Before the Drink Intake		Immediately after the Drink Intake		At 15 min after the Drink Intake		At 60 min after the Drink Intake	
	M	SD	M	SD	M	SD	M	SD
Energetic								
0-g milk group	3.29	(1.38)	3.33	(1.06)	3.29	(1.38)	3.57	(1.29)
10-g milk group	3.22	(1.35)	3.22	(1.40)	3.61	(1.42)	3.67	(1.57)
15-g milk group	3.39	(1.04)	3.72	(1.13)	3.11	(1.37)	3.50	(1.29)
Lively								
0-g milk group	0.29	(0.56)	0.14	(0.36)	0.43	(0.98)	0.24	(0.44)
10-g milk group	0.83	(0.99)	0.56	(0.98)	0.83	(0.79)	0.83	(1.04)
15-g milk group	0.39	(0.78)	0.11	(0.32)	0.61	(0.92)	0.72	(1.07)
Lethargic								
0-g milk group	1.19	(1.17)	0.71	(1.06)	0.90	(1.30)	0.95	(1.24)
10-g milk group	1.06	(1.11)	0.78	(0.81)	0.67	(0.77)	1.06	(0.87)
15-g milk group	1.50	(1.15)	1.00	(1.33)	1.06	(1.16)	1.33	(1.19)
Listless								
0-g milk group	1.43	(1.08)	1.62	(1.20)	1.76	(1.34)	1.81	(1.63)
10-g milk group	1.67	(1.61)	1.78	(1.40)	1.72	(1.13)	1.33	(1.08)
15-g milk group	1.11	(1.08)	1.94	(1.06)	1.71	(1.16)	1.33	(1.08)
Relaxed								
0-g milk group	3.29	(1.19)	3.33	(1.15)	3.10	(1.45)	3.38	(1.32)
10-g milk group	3.00	(1.41)	2.78	(1.52)	2.56	(1.34)	2.67	(1.53)
15-g milk group	3.17	(1.04)	3.44	(1.29)	2.94	(0.94)	3.11	(1.45)
Calm								
0-g milk group	0.48	(0.81)	0.44	(0.36)	0.29	(0.56)	0.48	(1.03)
10-g milk group	0.43	(1.15)	0.44	(0.70)	0.78	(0.81)	0.61	(1.09)
15-g milk group	0.44	(0.62)	0.42	(0.55)	0.44	(0.70)	0.39	(0.85)
Irritated								
0-g milk group	1.19	(1.33)	0.81	(1.03)	1.14	(1.39)	1.05	(1.28)
10-g milk group	1.28	(1.13)	1.28	(1.23)	1.11	(1.13)	1.44	(1.15)
15-g milk group	1.39	(1.33)	1.22	(1.35)	1.17	(1.15)	1.56	(1.25)
Nervous								
0-g milk group	1.33	(1.06)	1.81	(1.17)	1.81	(1.50)	1.67	(1.49)
10-g milk group	1.56	(1.46)	1.72	(1.36)	1.33	(1.24)	1.33	(1.03)
15-g milk group	1.39	(1.24)	1.83	(1.25)	1.67	(1.08)	1.22	(1.22)

Note: 0-g milk group = 0-g milk protein drink group, 10-g milk group = 10-g milk protein drink group, 15-g milk group = 15-g milk protein drink group, M = mean, SD = standard deviation.

## Data Availability

The datasets used and analyzed in the current study are available from the corresponding author upon reasonable request.

## Supplementary Materials

The following supporting information can be downloaded at: https://www.mdpi.com/article/10.3390/nu15020431/s1, Table S1: CONSORT Statement.
