# Supplementary material for: Acute Benefits of Acidified Milk Drinks with 10-g and 15-g Protein on Shifting and Updating Performances in Young Adults: A Randomized Controlled Trial
Source: Nutrients. 2023 Jan 13;15(2):431. doi: 10.3390/nu15020431 (PMC9860894; doi:10.3390/nu15020431)
Supplement: Supplementary file 1 [file nutrients-15-00431-s001.zip › nutrients-2130759-supplementary.pdf]

Table S1. CONSORT Statement.

CONSORT 2010 checklist of information to include when reporting a randomised trial *

Section/Topic	Item No	Checklist item	Reported on page No
**Title and abstract**			
	1a	Identification as a randomised trial in the title	Title
	1b	Structured summary of trial design, methods, results, and conclusions (for specific guidance see CONSORT for abstracts)	Abstract
**Introduction**			
Background and objectives	2a	Scientific background and explanation of rationale	Introduction
	2b	Specific objectives or hypotheses	Introduction
**Methods**			
Trial design	3a	Description of trial design (such as parallel, factorial) including allocation ratio	2.1
	3b	Important changes to methods after trial commencement (such as eligibility criteria), with reasons	n/a
Participants	4a	Eligibility criteria for participants	2.2
	4b	Settings and locations where the data were collected	2.1
Interventions	5	The interventions for each group with sufficient details to allow replication, including how and when they were actually administered	2.6 & 2.7
Outcomes	6a	Completely defined pre-specified primary and secondary outcome measures, including how and when they were assessed	2.8
	6b	Any changes to trial outcomes after the trial commenced, with reasons	n/a
Sample size	7a	How sample size was determined	2.4
	7b	When applicable, explanation of any interim analyses and stopping guidelines	n/a
Randomisation:			
Sequence generation	8a	Method used to generate the random allocation sequence	2.5
	8b	Type of randomisation; details of any restriction (such as blocking and block size)	2.5
Allocation concealment mechanism	9	Mechanism used to implement the random allocation sequence (such as sequentially numbered containers), describing any steps taken to conceal the sequence until interventions were assigned	2.5
Implementation	10	Who generated the random allocation sequence, who enrolled participants, and who assigned participants to interventions	2.5

Blinding	11a	If done, who was blinded after assignment to interventions (for example, participants, care providers, those assessing outcomes) and how	2.1
	11b	If relevant, description of the similarity of interventions	
Statistical methods	12a	Statistical methods used to compare groups for primary and secondary outcomes	2.13
	12b	Methods for additional analyses, such as subgroup analyses and adjusted analyses	2.13
**Results**			
Participant flow (a diagram is strongly recommended)	13a	For each group, the numbers of participants who were randomly assigned, received intended treatment, and were analysed for the primary outcome	3
	13b	For each group, losses and exclusions after randomisation, together with reasons	3
Recruitment	14a	Dates defining the periods of recruitment and follow-up	2.1
	14b	Why the trial ended or was stopped	n/a
Baseline data	15	A table showing baseline demographic and clinical characteristics for each group	Table1
Numbers analysed	16	For each group, number of participants (denominator) included in each analysis and whether the analysis was by original assigned groups	Figure1
Outcomes and estimation	17a	For each primary and secondary outcome, results for each group, and the estimated effect size and its precision (such as 95% confidence interval)	3
	17b	For binary outcomes, presentation of both absolute and relative effect sizes is recommended	
Ancillary analyses	18	Results of any other analyses performed, including subgroup analyses and adjusted analyses, distinguishing pre-specified from exploratory	n/a
Harms	19	All important harms or unintended effects in each group (for specific guidance see CONSORT for harms)	n/a
**Discussion**			
Limitations	20	Trial limitations, addressing sources of potential bias, imprecision, and, if relevant, multiplicity of analyses	4
Generalisability	21	Generalisability (external validity, applicability) of the trial findings	4
Interpretation	22	Interpretation consistent with results, balancing benefits and harms, and considering other relevant evidence	4
**Other information**			
Registration	23	Registration number and name of trial registry	Abstract
Protocol	24	Where the full trial protocol can be accessed, if available	n/a
Funding	25	Sources of funding and other support (such as supply of drugs), role of funders	Funding

* We strongly recommend reading this statement in conjunction with the CONSORT 2010 Explanation and Elaboration for important clarifications on all the items. If relevant, we also recommend reading CONSORT extensions for cluster randomised trials, non-inferiority and equivalence trials, non-pharmacological treatments, herbal interventions, and pragmatic trials. Additional extensions are forthcoming: for those and for up to date references relevant to this checklist, see www.consort-statement.org (accessed on 10 January 2023).
